# Overground vs. treadmill-based robotic gait training to improve seated balance in people with motor-complete spinal cord injury: a case report

**DOI:** 10.1186/s12984-017-0236-z

**Published:** 2017-04-11

**Authors:** Amanda E. Chisholm, Raed A. Alamro, Alison M. M. Williams, Tania Lam

**Affiliations:** 1 School of Kinesiology, University of British Columbia, Vancouver, BC Canada; 2 International Collaboration On Repair Discoveries, Vancouver Costal Health Research Institute, University of British Columbia, 818 West 10th Avenue, Vancouver, BC Canada V5Z 1M9

**Keywords:** Balance, Gait, Motor activity, Robotics, Spinal cord injury

## Abstract

**Background:**

Robotic overground gait training devices, such as the Ekso, require users to actively participate in triggering steps through weight-shifting movements. It remains unknown how much the trunk muscles are activated during these movements, and if it is possible to transfer training effects to seated balance control. This study was conducted to compare the activity of postural control muscles of the trunk during overground (Ekso) vs. treadmill-based (Lokomat) robotic gait training, and evaluate changes in seated balance control in people with high-thoracic motor-complete spinal cord injury (SCI).

**Methods:**

Three individuals with motor-complete SCI from C7-T4, assumed to have no voluntary motor function below the chest, underwent robotic gait training. The participants were randomly assigned to Ekso-Lokomat-Ekso or Lokomat-Ekso-Lokomat for 10 sessions within each intervention phase for a total of 30 sessions. We evaluated static and dynamic balance control through analysis of center of pressure (COP) movements after each intervention phase. Surface electromyography was used to compare activity of the abdominal and erector spinae muscles during Ekso and Lokomat walking.

**Results:**

We observed improved postural stability after training with Ekso compared to Lokomat during static balance tasks, indicated by reduced COP root mean square distance and ellipse area. In addition, Ekso training increased total distance of COP movements during a dynamic balance task. The trunk muscles showed increased activation during Ekso overground walking compared to Lokomat walking.

**Conclusions:**

Our findings suggest that the Ekso actively recruits trunk muscles through postural control mechanisms, which may lead to improved balance during sitting. Developing effective training strategies to reactivate the trunk muscles is important to facilitate independence during seated balance activity in people with SCI.

## Background

Rehabilitation for individuals with a spinal cord injury (SCI) mainly focuses on achieving functional independence in self-care and mobility [1]. The ability to maintain postural stability during sitting is important to perform daily functional activities [2]. However, the loss of normal postural synergies along with weakness or paralysis of the trunk muscles significantly impairs sitting balance [3]. Thus, improving seated postural stability is an important goal in most SCI rehabilitation programs.

For people diagnosed with motor-complete SCI above T6, it may be incorrectly assumed that they are unable to engage muscles of the trunk since established clinical methods (i.e. International Standards for Neurological Classification of Spinal Cord Injury, ISNCSCI) rely on sensory function in the thoracic spinal segments to determine the level and completeness of injury. Evidence from previous studies show that activity in the trunk muscles can be detected by manual palpation [4, 5], and electromyography (EMG) [6]. These methods have demonstrated the presence of abdominal muscle activity below the level of injury in individuals with motor-complete SCI [6, 7]. As well, the use of transcranial magnetic stimulation (TMS) has revealed motor-evoked potentials in the abdominal muscles below the level of injury for people classified as motor-complete SCI based on the ISNCSCI examination [7]. This implies some preservation of the corticospinal tract to the trunk muscles, raising the possibility for rehabilitation interventions to improve postural control and function in this sub-population.

Dynamic postural control is a well-known requirement for successful gait performance [8]. Robotic lower limb devices, such as the Lokomat, facilitate gait rehabilitation based on principles of body-weight support (BWS) treadmill training. However, it remains unknown how they engage postural muscles to control balance during standing and walking. Research in people with multiple sclerosis has demonstrated that gait training strategies that provide BWS may reduce the demand on postural muscles [9]. Gait training with the trunk passively supported by a harness implies less need for active dynamic stabilization. Consequently, the normal trunk muscle activity and movements that are important for the retention of posture and balance may not be involved in the gait training.

Advances in robotic technology, such as the Ekso™, allows for overground training and may provide a better opportunity for individuals with motor-complete SCI to engage the trunk muscles. The Ekso allows users to actively participate in the control of walking through subtle trunk motions to shift their weight over the appropriate foot in order to trigger each step. While this device is new to rehabilitation centers, current studies on a similar device have demonstrated safety and efficacy of use for gait training [10, 11].

It has also been demonstrated that training in one task (e.g. gait training) may transfer beneficial effects to other tasks [12, 13]. If training with robotics challenges dynamic postural control, secondary benefits of training may include the transfer of improved postural control to seated balance activities. Impaired motor and sensory functions after SCI can contribute to difficulty sitting unsupported, and compensatory patterns of muscle activation are often engaged to maintain postural support [3]. This is even more critical for individuals who are required to perform activities of daily living from a wheelchair, and need to be able to reach in all directions.

The purpose of this case study was to determine how postural control muscles of the trunk are challenged during different methods of robotic-assisted gait performance, and evaluate changes in seated balance control after gait training with robotics in people with motor-complete SCI above T6. If overground robotic-assisted gait training (i.e. Ekso) is a successful intervention to engage the trunk muscles compared to a treadmill-based method (i.e. Lokomat), we propose that increased limits of stability (LOS) during dynamic balance control and reduced COP sway during static balance control will be observed.

## Methods

Three participants who sustained a traumatic motor-complete SCI between C7-T4 (American Spinal Injury Association Impairment Scale; AIS A and B) 18–25 years ago (Table 1) volunteered for this study. All participants used a wheelchair for mobility and were independent in their activities of daily living. They had no significant medical history, and one participant was taking prescription medications for a bladder infection. Participants were able to maintain an unsupported seated posture for at least 1 min. They were free of using robotic gait devices (i.e. Lokomat and Ekso) for 1 year prior to the study. All participants provided written informed consent and all procedures were approved by the University of British Columbia Clinical Research Ethics Board.

**Table 1:** Participant demographic and clinical data

	P1	P2	P3
Age (y)	41	42	39
Weight (kg)	92.3	68.0	68.8
Height (cm)	183	170	178
Gender	M	M	F
Injury Level	T3	C7	T4
AIS	A	B	A
Post Injury (y)	23	18	25
UEMS (/50)	50	34	50
Pin Prick (/112)	40	62	43
Light Touch (/112)	41	67	43

*AIS* American Spinal Injury Association Impairment Scale, *UEMS* upper extremity motor score. Higher scores on pin prick and light touch scales of the ISNCSCI indicate better sensory function

### Gait training intervention

We used an alternating treatment design with three intervention phases to compare the Ekso and Lokomat methods of robotic gait training. The two groups were Ekso-Lokomat-Ekso and Lokomat-Ekso-Lokomat, with 10 training sessions in each intervention phase for a total of 30 sessions and no washout between intervention phases. Randomized allocation of the participants to the groups was concealed at the examination. Seated balance control outcome measures were repeated at the end of each intervention phase.

Participants performed up to 45 min of robotic-assisted gait training 3–4 times per week. Rest breaks were provided when needed by the participant. Participants reported their rating of perceived exertion (RPE) on the Borg CR-10 Scale every 10 min during training [14].

Initial training with the Ekso™ (Ekso Bionics, California, USA) consisted of sit-to-stand, standing balance, weight shifting, and stand-to-sit functions. Training focused on improving walking performance (triggering steps, executing a turn, and stopping). All participants were in ‘ProStep’ mode, wherein steps are automatically triggered when the weight-shifting targets are achieved, within the first session. Auditory cues for the weight-shifting targets were provided as feedback, and removed when an efficient gait pattern was maintained. The 10-meter walk test (10MWT) was used to record their fastest possible gait speed during training, which will reflect their ability to weight shift and trigger steps efficiently. The best time of three trials was recorded. For each session, we documented ‘Up Time’ (combined standing and walking time), ‘Walk Time’ (walking time only), and the number of steps taken.

Gait training with the Lokomat robotic gait orthosis focused on increasing gait speed. Treadmill speed was set to the fastest speed that the patient could tolerate, and subsequently increased by increments of 0.1 km/h every 10 min. If spasticity was exaggerated or poor foot contact with the treadmill was observed, the speed was lowered by 0.1 km/h. The level of BWS was adjusted to the minimum tolerated by the patient while ensuring appropriate stance phase kinematics. For each session, we documented BWS, walking speed and total distance.

### Seated balance control

**Fig. 1:**
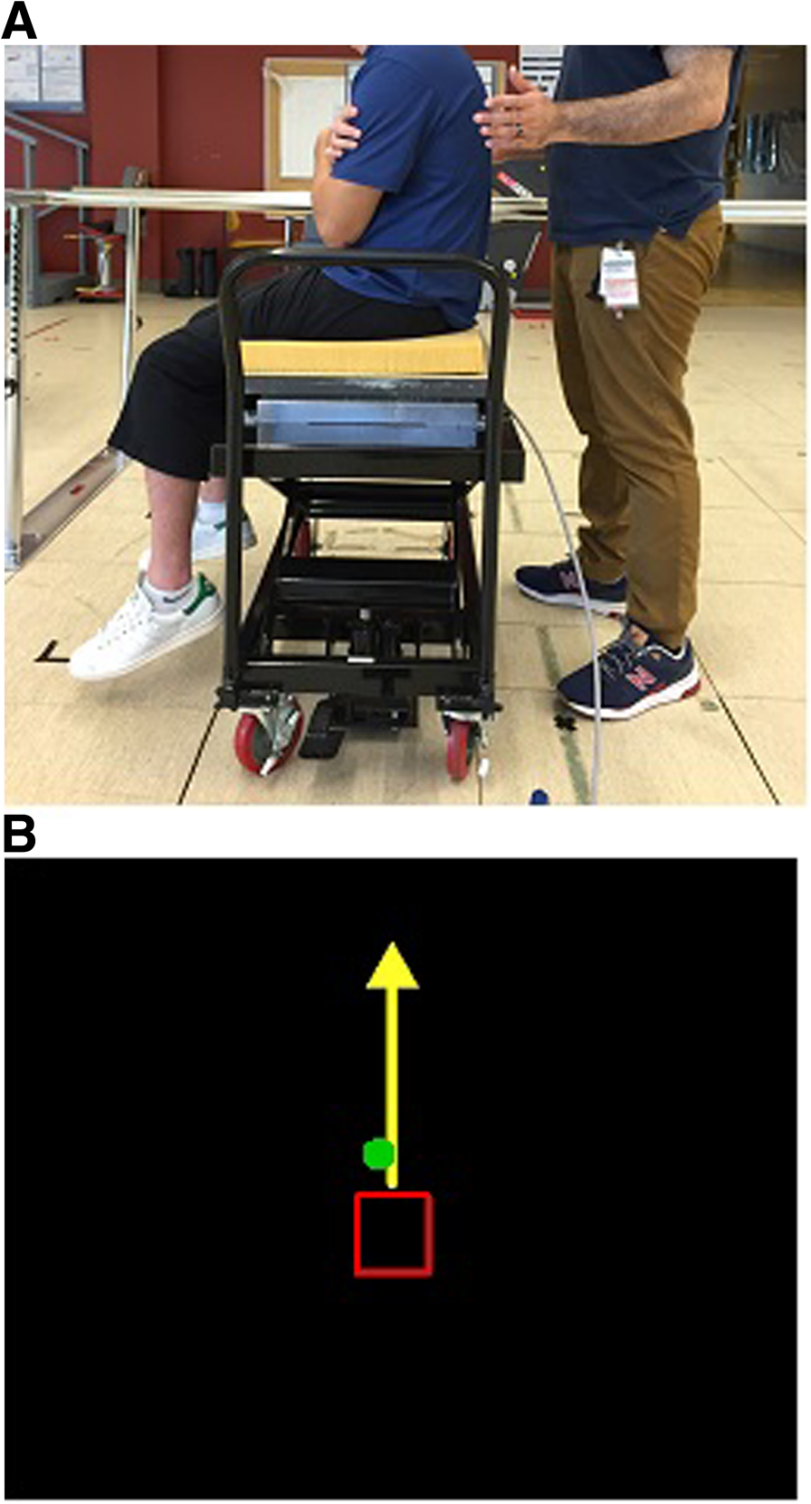
A picture of the seated balance control measurement setup; **a** participant is seated on the forceplate with feet off the ground, and **b** the computer monitor displays the limits of stability test (COP position – *green dot*, baseline limit – *red box*, movement direction – *yellow arrow*)

Two baseline assessments of seated balance control were conducted prior to training and separated by 1 week. The assessment was repeated within 1 week of the final training session at the end of each intervention phase. We evaluated seated balance control by asking participants to sit on a forceplate (Bertec; Columbus, Ohio) covered with a foam pad. The forceplate was elevated so that the feet were off the ground. Participants were instructed to sit as still as possible while performing two static sitting balance tasks (quiet sitting with eyes open and with eyes closed) for 60 s with their arms crossed at the chest (Fig. 1a). During the eyes open task, individuals focused their gaze on a target 10 ft away. This protocol has been previously used to verify impaired seated postural control in people with SCI [15]. We also performed a dynamic sitting task to evaluate their LOS in the eight cardinal directions. During the LOS test, each trial started with 20 s of quiet sitting with eyes open to establish a baseline limit, calculated as the COP mean position plus four times the standard deviation (SD). Then, we provided visual biofeedback of their COP position (e.g. green dot) and baseline limits (e.g. a red box scaled to the baseline limit and centered at the mean COP position) on a computer monitor. Participants were instructed to lean as far as they could without losing their balance and return their COP within the baseline limit (Fig. 1b). There was no time constraint to complete the movement and the order of each direction was randomized. The movement direction was presented as a yellow arrow on the monitor. Two trials were recorded for each static balance task and the LOS test. This assessment was repeated 1 week later to establish a stable baseline. Data were collected at 100 Hz and stored for offline analysis.

These data were subsequently used to calculate the root mean square distance (RDIST) and velocity (RVEL) from the mean COP position to examine overall postural stability and the amount of postural activity during the static balance tasks [16]. We also calculated a 95% confidence ellipse area (AREA-CE) during static balance to measure stability performance [16]. The LOS test was scored as the total distance (sum of the maximum distance between the COP mean position and the furthest point in each direction). A mean was calculated per condition for all outcome measures.

Participants also performed two clinical measures of seated balance function at baseline and after each intervention phase. The T-shirt Test measures the time taken by the participants to don and then doff a T-shirt. The mean of two trials was calculated with the total time. For the Modified Functional Reach Test (mFRT), participants sat unsupported with their hips, knees, and ankles at 90°, then pushed a ruler forward with both arms and held their maximum position for at least 2 s. The mean of three trials was calculated with the distance. These tests of unsupported sitting have been proven reliable and valid in SCI [17, 18].

### Gait assessment

When participants were able to walk overground with the Ekso in ‘ProStep’ mode at a gait speed of at least 1.0 km/h, which is the minimum speed of the Lokomat, we conducted an assessment of trunk muscle activity comparing each method of robotic-assisted gait. EMG data were recorded using surface electrodes (SX230-1000, Biometrics Ltd., Newport, UK) connected to a portable data acquisition system (DataLOG, Biometrics Ltd., Newport, UK). Electrodes were placed on the right side for the rectus abdominis (RA)–3 cm lateral and 2 cm caudal to umbilicus; external oblique (EO)–2 cm below the lowest point of the rib cage [6]; and erector spinae (ES)–2 cm lateral to the T3, T12 and L4 spinous processes [19]. EMG signals were recorded at 1000 Hz. Foot switches were used to determine heel strike and toe off for each step. Participants performed or attempted a maximum voluntary contraction (MVC) at the trunk for flexion, lateral flexion, rotation and extension. Baseline EMG activity for all muscles was recorded while participants were lying supine.

Participants performed two walking conditions at matched speeds: 1) Ekso and 2) Lokomat. Three trials were recorded per condition. Overground speed was calculated from the time taken to traverse the middle 10 m of the 12 m path. During Lokomat walking, we used a standardized BWS level set at 50% of the participant’s body weight.

The average EMG amplitude recorded during MVC was used for normalization. EMG data was filtered with a sixth-order dual pass Butterworth filter at a high-pass of 30 Hz to remove electrocardiography artifact, then rectified and filtered with a sixth order dual pass Butterworth filter at a low pass of 50 Hz using custom routines written in MATLAB (Mathworks, Natick, MA, USA). Data were separated into steps synchronized to right heel strike. The average time series amplitude of at least 20 steps was calculated per condition. Then, the root mean square (RMS) of the EMG signal over each gait cycle for each muscle was calculated to quantify the EMG amplitude of the trunk muscle activity. RMS was also calculated for the baseline EMG activity for all muscles during static supine position.

## Results

**Fig. 2:**
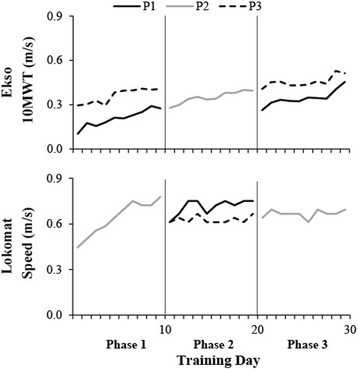
Gait speed is plotted for each training session per participant (P1 – *solid black line*, P2 – *solid grey line*, P3 – *dotted black line*). Gait speed was determined by the 10MWT for Ekso training, and the maximum speed achieved during Lokomat training

P1 and P3 were randomized to Ekso-Lokomat-Ekso, while P2 trained in the Lokomat-Ekso-Lokomat group. All participants were able to physically tolerate robotic-assisted gait training; all sessions were completed and no adverse events occurred. RPEs reported ranged from 2 to 8 for Ekso training and 0.5–6 for Lokomat training. Figure 2 shows the progression of gait speed over each phase of training.

### Static balance control

**Fig. 3:**
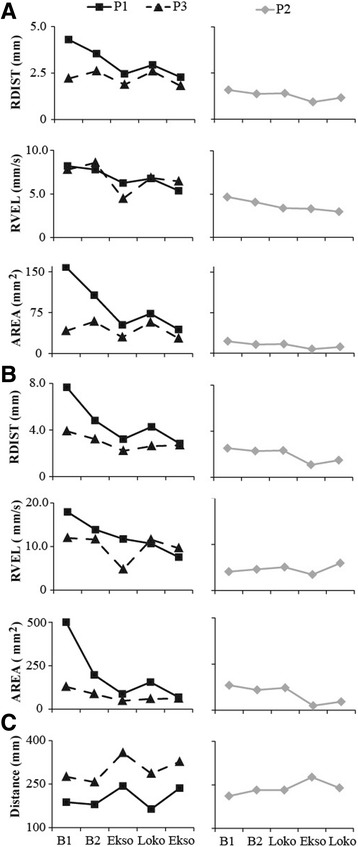
COP outcome measures are plotted for baseline 1, baseline 2, and post each intervention phase; mean COP RDIST, RVEL and AREA-CE of the static balance tasks (**a** eyes open and **b** eyes closed), and **c** mean total distance of the dynamic balance task

Baseline postural sway measures indicate that participants had greater instability during eyes closed compared to eyes open (Fig. 3a & b). During the first baseline assessment, static seated balance control with eyes closed was difficult for P1, who lost his balance and had to grab a handrail for recovery. Data on this trial was analyzed prior to the handrail recovery. All other trials were completed successfully.

After the first intervention phase of Ekso training, P1 and P3 reduced the RDIST, RVEL, and AREA-CE of the COP during both EO (Fig. 3a) and EC (Fig. 3b) conditions, indicating improvement in quiet sitting performance. After Lokomat training, there was a general increase in RDIST and AREA-CE, indicating worsening of performance. This was followed by general improvement in these measures (decreased scores) after the final Ekso training phase, except for the EC condition with P3. P1 continued to reduce RVEL across the Lokomat and final phase of Ekso training, while it increased back to baseline values for P3. The RDIST and AREA-CE remained similar for P2 between both baseline assessments and after the first intervention phase of Lokomat training in both EO and EC conditions. P2 reduced RDIST and AREA-CE after Ekso training compared to baseline and Lokomat training, with a larger effect in the eyes closed condition. RVEL showed no change across assessments for P2 with EO, but an increasing trend for EC with the exception of a slight decrease after Ekso training.

### Dynamic balance control

The change in LOS total distance showed a similar trend for P1 and P3: increase after Ekso training compared to baseline (indicating improved performance), then decrease after Lokomat training, and subsequent increase after the final Ekso training (Fig. 3c). P2 showed no change between baseline 2 and Lokomat training for LOS total distance, followed by an increase after Ekso training, and then a decrease after the second Lokomat training phase (Fig. 3c).

### Clinical measures of seated balance control

All participants slightly reduced their total time during the T-Shirt Test and increased their distance during the mFRT after Ekso training (Table 2). There was a tendency for greater time taken on the T-Shirt Test and shorter distance during the mFRT after Lokomat training as compared to Ekso training (Table 2).

**Table 2:** Summary of clinical measures of seated balance control

	T-Shirt Test				mFRT			
	B1	Ekso	Loko	Ekso	B1	Ekso	Loko	Ekso
P1	10.9	9.5	10.9	9.0	5.2	5.7	4.3	5.7
P3	15.1	10.0	10.8	10.2	4.2	9.3	6.7	13.0
	B1	Loko	Ekso	Loko	B1	Loko	Ekso	Loko
P2	17.9	14.7	12.9	13.4	3.3	6.3	9.0	6.0

*B1* first baseline assessment, *mFRT* Modified Functional Reach Test

### Trunk EMG during robotic-assisted gait

**Fig. 4:**
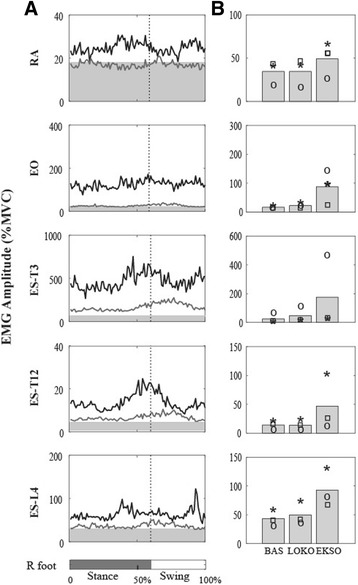
**a** the normalized muscle activity patterns of the rectus abdominis (RA), external oblique (EO), and erector spinae (ES) muscles are plotted over the gait cycle for P1 for all conditions; Ekso (EKSO –*black line*), and Lokomat (LOKO – *grey line*). The baseline activity (BAS – *light grey shaded* area) recorded during quiet lying is also displayed. **b** the average RMS amplitude across participants is plotted as a bar for each condition. Individual data from each participant is also displayed (P1 – *circle*, P2 – *square*, P3 – *star*)

The RA and EO muscles showed tonic activity over the gait cycle during Ekso and Lokomat walking, while the ES muscles showed a burst of activity at the transition from stance to swing that is more noticeable in the Ekso condition (Fig. 4a). Ekso walking produced higher trunk muscle activity compared to Lokomat walking in all participants (Fig. 4b). In fact, mean EMG amplitude in all muscles during Lokomat walking was similar to that recorded during baseline.

## Discussion

Advances in robotic-gait technology provide an exciting opportunity to explore possible training benefits to individuals with motor-complete SCI who are otherwise unable to practice standing and walking independently. Prior to training, we observed postural instability during seated balance that is consistent with previous reports in SCI [15, 20]. This case report demonstrates how overground robotic gait training with the Ekso engages the trunk muscles and could elicit training effects on static and dynamic seated balance control in people with high-thoracic motor-complete SCI. Conversely, after training with the Lokomat, a robotic-gait system that limits trunk movement, participants demonstrated no change in seated balance control. This is supported by our observations that RMS amplitudes of trunk muscle activity during Lokomat walking did not differ much from that recorded while lying supine. These results indicate that overground robotic gait training presents a unique strategy to re-activate muscles of the trunk to enhance postural stability, which is important for performing functional activities in a seated posture after SCI.

An important finding demonstrated by our study participants was the recruitment of trunk muscle activation while walking in the Ekso, even though they were classified as motor-complete above T6. Previous studies have implemented neurophysiological assessments to show sparing of the corticospinal tract below the level of injury in people classified as motor-complete [6, 7]. Although, we did not specifically evaluate preservation, participants did show greater activity during the MVCs compared to resting in a supine position. In addition, the Ekso engaged the trunk muscles considerably above resting levels and this may have produced a training effect.

We propose that the Ekso engages the trunk muscles through weight-shifting movements that are required to position the body over the appropriate foot to initiate a step. In able-bodied individuals, abdominal and back muscles are activated during walking and contribute to maintaining postural stability by producing angular accelerations at the trunk in the frontal and sagittal planes [21, 22]. During Ekso walking, we observed similar activation patterns of the RA, EO and ES as reported in able-bodied subjects who walked overground at a similar speed [23]. Increased activity in the EO may represent greater demands for lateral stabilization during trunk shifts. However, in the Lokomat, muscle activity levels were similar to those during static posture. It appears that the BWS may decrease or eliminate the need to produce angular accelerations for postural stability. A previous study reported reduced postural control demands as a result of limited trunk acceleration during gait with BWS in able-bodied adults [24]. Moreover, the percentage of the BWS provided by the Lokomat may affect trunk muscle activity. Trunk muscle activity recorded from able-bodied subjects and individuals with multiple sclerosis while walking on a treadmill supported by a harness showed increased activity of EO and decreased activity of ES as BWS percentage increased [9].

We observed evidence of improved seated balance control during static and dynamic tasks after training with an overground robotic exoskeleton. The transfer of improved balance control from standing to sitting is consistent with other studies showing transfer between gait and balance functions [12, 13]. Although we did not test this directly, it is possible that participants learned to use sensory cues from the body during gait training to improve balance while sitting. By providing spatial auditory biofeedback for the lateral and forward weight-shifting movements while using Ekso, the participants may have become more aware of their body’s position in space through improved sensorimotor integration [25, 26]. Furthermore, as training is progressed, the auditory cues are removed as long as the participant can maintain an efficient gait pattern. The reduced reliance of the auditory feedback along with the findings of improved seated balance following the Ekso phases are supportive of a learning effect, although further study is required to confirm this hypothesis.

In addition, postural control training in a standing posture may offer secondary benefits aimed at overcoming other health problems, such as bladder infections [27], spasticity [28], blood pressure homeostasis [29], and bone demineralization [30]. Thus practicing in a standing posture may decrease the risk of secondary complications and subsequently improve the quality of life of individuals with SCI. Since seated balance plays an important role in performance of daily activities [31], developing effective training strategies to enhance postural control and facilitate prevention of secondary complications from prolonged sitting is important to health and quality of life for people with SCI.

This case report provides important information comparing the possible effects of different robotic-gait training methods; however there are several limitations that need to be addressed. We did not provide a wash-out period between interventions to eliminate the possibility of carry-over effects, and determine retention of the changes in seated balance control. For example, the positive training effects from using the Ekso may have been maintained into the subsequent Lokomat intervention phase. We used a double baseline prior to training to determine stability in the COP outcome measures, however P1 showed better balance control at the second baseline as he had to use a hand rail to prevent falling during eyes closed during the first assessment. Also, it is possible that wearing a harness and the suspension system has an influence on muscle activity during gait due to a change in trunk posture [24]. The BWS system used in this study has four suspension points, which may further reduce postural stability demands during gait training compared to other systems that only have one or two suspension points. Hence, the results of this study cannot be extrapolated to other suspension systems. In addition, our EMG assessment provided only a cross-sectional view of enhance trunk muscle activity during Ekso compared to Lokomat walking. Further study with a larger sample is required to evaluate the training effect of Ekso walking on trunk muscle activity, and to confirm the clinical significance of the changes in seated balance control.

## Conclusions

In summary, this case report has shown that overground robotic gait training in the Ekso enhances trunk muscle activity relative to Lokomat, which may lead to improve seated postural control in motor-complete SCI. These findings raise interesting possibilities for gait rehabilitation strategies for people with high-thoracic motor-complete SCI. These results emphasize the importance of trunk control in sitting balance, and indicate the importance of recovering trunk function in rehabilitation of individuals with SCI as an approach to improve their sitting balance required for functional activities.

## Abbreviations

10MWT: 10-meter walk test
AIS: American Spinal Injury Association Impairment Scale
AREA-CE: Confidence ellipse area
BWS: Body-weight support
COP: Centre of pressure
EMG: Electromyography
EO: External oblique
ES: Erector spinae
ISNCSCI: International Standards for Neurological Classification of Spinal Cord Injury
LOS: Limits of stability
mFRT: Modified Functional Reach Test
MVC: Maximum voluntary contraction
RA: Rectus abdominis
RDIST: Root mean square distance
RMS: Root mean square
RPE: Rating of perceived exertion
RVEL: Root mean square velocity
SCI: Spinal cord injury
